# Chronological versus skeletal age and its relationship with motivational profiles and psychological skills among male youth football players from South Africa

**DOI:** 10.17159/2078-516X/2025/v37i1a21882

**Published:** 2025-12-15

**Authors:** SR Dube, EH Wik, SP Cumming, EW Derman, HW Grobbelaar

**Affiliations:** 1 Division of Sport Science, Department of Exercise, Sport and Lifestyle Medicine, Faculty of Medicine and Health Sciences, Stellenbosch University, Stellenbosch, South Africa; 2 Division of Sport and Exercise Medicine, Department of Exercise, Sport and Lifestyle Medicine, Faculty of Medicine and Health Sciences, Stellenbosch University, Stellenbosch, South Africa; 3 Department for Health, Faculty of Humanities and Social Sciences, University of Bath, Bath, United Kingdom; 4 International Olympic Committee (IOC) Research Centre, South Africa; 5 Aspetar Orthopaedic and Sports Medicine Hospital, Doha, Qatar

**Keywords:** adolescent football, self-determined motivation, psychological skills, biological maturation

## Abstract

**Background:**

Self-determined motivation (SDM) and sport psychological coping skills are important for developing footballers.

**Objectives:**

This study examined relationships between chronological age (CA), skeletal age (SA), and psychological variables among South African youth players.

**Methods:**

Data were collected from 109 male players across three football academies (mean CA: 14.8±2.4 years, mean SA: 14.2±2.1 years). Participants completed the Sport Motivation Scale-II (SMS-II) and the Athletic Coping Skills Inventory-28 (ACSI-28).

**Results:**

Amotivation, external, and introjected regulations differed significantly across CA groups, negatively affecting the Relative Autonomy Index (RAI). SDM, as reflected by the RAI, declined with CA, reaching its lowest point between 15 and 17 years. SA grouping revealed significant differences for amotivation and external regulation. Coachability differed across CA and SA groups. Amotivation, external regulation, and coachability showed positive correlations with CA, while amotivation and coachability showed positive correlations with SA. Concentration showed opposite correlations with CA and SA when controlling for the other variable.

**Conclusion:**

CA grouping appeared to reflect differences in SDM more effectively than SA. While some psychological and motivational variables aligned more closely with CA or SA, others did not, highlighting the complex and multifactorial nature of these developmental relationships. Given the low internal consistency of several subscales, these findings should be interpreted cautiously and viewed as exploratory. Future research should adopt longitudinal designs and utilise culturally appropriate, psychometrically robust tools better to understand the development of psychological skills in this population.

Sport success extends beyond physical and technical prowess; it also involves the integration of psychological characteristics during both practice and competition.^[1–3]^ Psychological characteristics can enhance performance, especially when players are evenly matched physically and technically, but are often neglected when compared to technical, tactical, and physical factors.^[1,4–5]^ For example, Salmon et al.^[6]^ highlight the importance of imagery in competition, particularly at the elite level. Concentration is another psychological factor that differentiates players as they advance to higher competitive levels.^[4]^ A study on African youth football players found that concentration distinguished higher-ranked teams from lower-ranked ones, whilst none of the other psychological skills showed significant differences.^[4]^ Furthermore, psychological skills are integral to long-term athletic development.^[1–3,5]^

In youth football, players are usually grouped into one- or two-year age bands, resulting in chronological age (CA) variations of up to two years.^[7–9]^ However, skeletal age (SA) - an indicator of biological maturity - can differ by as much as five years among individuals of the same CA.^[7,8,10]^ Such biological maturation differences influence not only physical performance but also the development of psychological skills and motivational profiles.^[3,8,9,11]^ Sport motivation can be explained by the Self-Determination Theory (SDT), which categorises motivation into different forms of regulation (intrinsic, integrated, identified, introjected, extrinsic, and amotivation) based on the degree of self-determination.^[12–14]^ SDT postulates that fulfilling the needs for autonomy, relatedness, and competence fosters more self-determined motivation (SDM).^[13,14]^

Early-maturing athletes often report higher extrinsic motivation and more positive self-perceptions, likely due to the physical advantages they experience during adolescence.^[9,15,16]^ Conversely, late-maturing athletes tend to develop stronger psychological resilience and engage in self-regulated learning more often, thereby compensating for physical disadvantages through well-developed cognitive and emotional attributes.^[8,9,16]^ The “underdog hypothesis” posits that late-maturing athletes must possess and/or develop adaptive psychological skills to overcome their physical and functional limitations, thereby potentially contributing to long-term sporting success.^[8,15]^ Long-term success will, however, only be achieved if these players are retained in the academy system. Still, evidence suggests that late-maturing players are mainly absent from academy football by the age of 15.^[8]^

In support of the underdog hypothesis, a recent study involving male football players from 10 African countries found that late-maturing players scored significantly higher in coping with adversity, as well as in goal-setting and mental preparation, than early-maturing players.^[3]^ Despite these strengths, talented late-maturing athletes are more likely to be deselected and/or drop out, often turning to other sports in which they may achieve greater success and fulfilment.^[9,16]^ Furthermore, youth football players exhibit fluctuating motivation levels influenced by their age, with a notable decline in SDM as players transition from the U15 to U17 age-groups.^[12]^ This decline in SDM is suggested to be driven by the external rewards associated with pursuing professional playing status.^[12]^ Despite this, youth elite athletes consistently demonstrate higher levels of SDM than their non-elite counterparts, highlighting its essential role in athletic performance.^[12]^ These findings highlight the important role of motivation in sustaining sport participation and achievement.^[12,13,16]^

In this study, we aim to explore the relationship between CA and SA and self-determined motivation and coping skills in South African male adolescent football players. Specifically, how do psychological characteristics differ between younger and older players, and what role do CA and SA play in shaping these attributes? This focus is especially relevant in South African youth football, where limited research and support exist regarding the development of psychological skills.^[3,4]^ The results may inform coaching strategies and development programs to support the holistic development of young athletes.

## Methods

This study received ethical clearance from Stellenbosch University, Health Research Ethics Committee 1 (HREC No: S23/10/250) to use de-identified data from an existing database (B22/02/001) with prior parental consent and player assent. A cross-sectional, quantitative design was employed to investigate the associations between participants’ CA and SA, and various psychological variables, allowing for comparisons of the variables across age-groups as well as correlations between the variables of interest.

### Participants

The sample included 109 adolescent male football players from three football academies in the Western Cape Province of South Africa. All players trained 3–4 times per week under qualified coaches and competed in a structured two-year age-group league (e.g., U12, U14, U16, U18). Two academies were affiliated with clubs that compete in South Africa’s first division, Premier Soccer League, whilst the other academy was associated with a club from the third division. The sample’s mean values for CA and SA (mean±standard deviation) were 14.8±2.4 years and 14.2±2.1 years, respectively.

### Measures

CA was expressed as a decimal number and calculated by subtracting each participant’s birthdate from the date of assessment. Biological maturation was assessed using the BAUSport™ system (SonicBone Medical Ltd., Israel), a quantitative ultrasound tool that provides a radiation-free alternative to traditional X-ray methods.^[10,17]^ The BAUSport™ system estimates SA by evaluating three sites: the distal radius and ulna, the metacarpals, and the proximal third phalanx,^[17]^ requiring information about an individual’s sex, date of birth, height and body mass.^[10,17]^ The system has previously demonstrated a high degree of concordance and reproducibility in the estimation of bone age when compared to established estimates of SA using radiographs of the hand-wrist.^[10, 17]^ Percentile-based age-groups were created for the full sample using both CA and SA (CA/SA1: 0–25^th^ percentile, CA/SA2: 26^th^–50^th^ percentile, CA/SA3: 51^st^–75^th^ percentile, CA/SA4: 76^th^–100^th^ percentile). This method enabled comparisons between relatively younger/older (CA) players and those who were less/more biologically mature (SA) across the entire cohort, rather than within specific playing levels or CA categories. This approach reflects the typical two-year league format and ensures balanced group sizes for meaningful statistical comparisons.

Questionnaires were completed at the respective clubs from August to November 2023, during the in-season period, which coincided with the same period in which SA assessments were conducted. Players completed the questionnaires individually, using paper and pen, before training sessions in an indoor setting with minimal distractions. At least three research assistants were present to provide guidance. All 109 participants completed the Sports Motivation Scale II (SMS-II),^[14]^ while 107 completed the Athletic Coping Skills Inventory-28 (ACSI-28).^[18]^ The English versions of both the SMS-II and ACSI-28 were used due to the absence of validated translations or standardisations in any of the other 10 official South African languages.

The SMS-II consists of 18 items assessing six motivational profiles: amotivation, extrinsic motivation (external, introjected, and identified regulation), integrated regulation, and intrinsic motivation.^[14]^ Participants rate their agreement with reasons for sports participation on a 7-point Likert scale (1: Not at all true, 4: Somewhat true, 7: Very true). Each subscale consists of three items, with scores ranging from 3 to 21, where higher scores indicate stronger endorsement of the respective motivational type. The Relative Autonomy Index (RAI) quantifies perceived self-determination through a weighted calculation of the six subscale scores: RAI = (Intrinsic × 3) + (Integrated × 2) + (Identified × 1) + (Introjected × −1) + (External × −2) + (Amotivated × −3).^[13,14]^ The ACSI-28 measures seven psychological coping skills: coping with adversity, coachability, concentration, confidence, goal setting and mental preparation, peaking under pressure, and freedom from worry.^[18]^ The inventory includes 28 items, with participants rating each item on a 4-point Likert scale. Subscale scores range from 0 to 12, with higher scores indicating stronger coping strategies. The total ACSI-28 score, ranging from 0 to 84, reflects the overall coping level.^[18]^

### Statistical analysis

All statistical analyses were performed using RStudio (version 2024.09.0). The results, along with the reproducible code, are available upon reasonable request. Descriptive statistics were used to summarise participants’ anthropometric data (body mass, height, BMI), SA and CA, and psychological characteristics. Means and standard deviations (SD) were calculated for continuous variables, while medians and interquartile ranges (IQR) describe the Likert-scale summed subscale data. Internal consistency of the psychological subscales was assessed using Cronbach’s alpha, with α≥0.70 considered acceptable. The Spearman-Brown formula was applied to assess how the reliability indices might improve if additional items were added to the respective instruments to determine the psychological subscales, resolving the low alpha values observed in some subscales. To compare paired measurements of CA and SA, the Wilcoxon signed-rank test was used. The test statistic (V) represents the sum of signed ranks for differences between paired observations. A rank biserial correlation was calculated to estimate the effect size.

The Kruskal-Wallis test was used to compare percentile-based age-groups (CA and SA). Effect sizes for overall group differences were calculated using epsilon squared (ɛ^2^), interpreted as negligible (<0.01), small (0.02–0.06), medium (0.07–0.14), or large (≥0.15). Post-hoc comparisons were conducted using the Wilcoxon rank-sum test. Given the exploratory aims and separate subscale analyses, unadjusted p-values were reported to highlight group differences. Pairwise effect sizes were calculated using rank biserial correlation (r_rb_), interpreted as small (0.10–0.24), medium (0.25–0.49), or large (≥0.50). Spearman’s rank correlation coefficients were computed to examine linear relationships between the age variables (CA and SA) and summed psychological scores. Partial correlations controlled for one age predictor to assess the unique contribution of the other. Correlation strength was classified as weak (0.00–0.30), moderate (0.31–0.50), or strong (>0.50). A significance level of p<0.05 was set throughout.

## Results

The Cronbach’s alpha values for the overall composite scores of the SMS-II (α=0.74, CI: 0.66–0.79) and ACSI-28 (α=0.73, CI: 0.63–0.79) indicated acceptable levels of internal consistency, while the individual subscale reliabilities varied from α=0.23 to 0.68. Table 1 presents the reliability statistics of the psychological subscales from the SMS-II and ACSI-28, showing that doubling the number of items for each subscale would improve the reliability of nine subscales to acceptable levels (≥0.70). The low reliability coefficients observed for some subscales require caution when interpreting results at the individual subscale level.

The mean SA was lower than the mean CA, indicating overall delayed maturation. A Wilcoxon signed-rank test confirmed this as a statistically significant difference: V=4448.0, p<0.001, with a medium rank biserial effect size of 0.48 (95% CI: 0.30 to 0.63). Within the sample, players in CA1 were predominantly classified as SA1 (n=23), with a smaller number in SA2 (n=6). In CA2, players were distributed across SA1 (n=6), SA2 (n=15), and SA3 (n=2). In CA3, the distribution included SA2 (n=5), SA3 (n=16), and SA4 (n=7), while CA4 had players in SA2 (n=3), SA3 (n=10), and SA4 (n=16). Table 2 presents descriptive statistics for the full sample. Introjected regulation (SMS-II) had the lowest mean, while intrinsic and identified regulation scored highest. The RAI showed high variability. Coachability, as well as confidence and achievement motivation (ACSI-28) had the highest means, while freedom from worry had the lowest, highlighting differences in motivation and coping skills. However, as noted earlier, some of these subscales demonstrated poor internal consistency, and results should be interpreted cautiously.

Table 3 presents comparisons across CA percentile groups. Among the SMS-II subscales, amotivation differed significantly (p<0.001, large ɛ^2^=0.15), with CA1 scoring lower than CA3 and CA4. External regulation (p<0.001, medium ɛ^2^=0.12) was lower in CA1 than CA3, while introjected regulation (p=0.02, medium ɛ^2^=0.10) differed between CA1 and CA3, and between CA3 and CA4. The RAI (p=0.01, medium ɛ^2^=0.10) was lower in CA3 than CA1 and CA4. Among the ACSI-28 subscales, only coachability differed significantly (p=0.04, medium ɛ^2^=0.06), with CA1 scoring lower than CA3 and CA4.

Table 4 presents comparisons across SA percentile groups. Among the SMS-II subscales, amotivation differed significantly (p<0.001, large ɛ^2^=0.15), with SA1 scoring lower than SA2, SA3, and SA4. External regulation (p<0.001, medium ɛ^2^=0.12) was lower in SA1 than SA2 and SA3, while SA3 scored higher than SA4. No differences were observed for the remaining SMS-II subscales or the RAI. As with the CA comparisons, coachability was the only ACSI-28 subscale showing significant group differences (p=0.02, medium ɛ^2^=0.10), with SA1 scoring lower than all other groups. Detailed pairwise comparisons and effect sizes are presented in Tables 5 (SMS-II) and 6 (ACSI-28).

Table 7 presents Spearman correlations. Amotivation was positively correlated with CA and SA, indicating that older and more mature players reported higher levels of amotivation (i.e., a lack of intent to act).

External regulation exhibited a weak positive correlation with CA but was not significantly associated with SA. The RAI was negatively associated with SA, suggesting lower SDM in more biologically mature players. Coachability was positively correlated with both CA and SA. Concentration showed opposing relationships with CA and SA; a positive association with CA when controlling for SA, and a negative association with SA when controlling for CA. While some group differences and correlations were statistically significant, these findings should be interpreted with caution where subscales had suboptimal internal consistency.

## Discussion

The study aimed to investigate relationships among chronological age, maturation, and psychological characteristics in adolescent male football players from the Western Cape Province of South Africa. The findings reveal that, on average, the players in this sample were late maturing, a departure from the early-maturation bias often observed in European and other youth academies.^[7,8]^ While certain motivational profiles and psychological skills were distinctly associated with CA or SA, other subscales did not show significant relationships, underscoring the nuanced ways in which CA and SA are related to these psychological variables. Although both the SMS-II and ACSI-28 have previously been used in South African samples, the internal consistency of several subscales was notably weak in the current sample. These tools may not have been fully suitable for this context or population, potentially due to a combination of internal factors (e.g., item phrasing, scale structure) and external influences (e.g., age, cultural relevance, contextual interpretation by participants). As a result, the understanding of subscale-level findings is limited, and conclusions regarding specific psychological constructs should be viewed as tentative.

Amotivation reflects a lack of intent to act. In contrast, external regulation is driven by rewards or the avoidance of punishment, and introjected regulation stems from internal pressures, such as guilt or a desire to seek approval.^[13,14]^ These motivations lie at the lower end of the self-determination continuum and negatively affect SDM, as expressed by the RAI.^[13]^ The observed differences in these subscales across age-groups suggest a decline in SDM as players age, with older players increasingly relying on controlled forms of motivation. Additionally, correlations suggest that both CA and SA are associated with a decline in motivation as players age and mature. Furthermore, the weak negative correlation between SA and RAI suggests that as players’ SA increases, their SDM decreases slightly. This finding aligns with previous research in the United Kingdom, which showed a decrease in SDM as players transition from U15 to U17.^[12]^ This shift in SDM is potentially linked to greater external pressures and expectations regarding professional opportunities.^[12]^ In South African football, players beyond the U19 category are typically expected to seek professional contracts in the South African Premier League or with international clubs, which may contribute towards a shift to less adaptive forms of motivation.

The shift in SDM suggests that changes in motivation across age-groups are not strictly linear, with distinct variations observed in SDM. Players in the youngest CA group (9.9–12.7 years) showed the highest SDM, while players in the CA2 group (12.7–14.9 years) exhibited a decrease in SDM, with the lowest observed in the CA3 group (14.9–16.7 years). Interestingly, the oldest group, CA4 (16.7–18.9 years), showed a rebound in SDM, indicating a fluctuation rather than a consistent decline. When grouped by SA, significant differences were found only for amotivation and external regulation, suggesting that CA may better capture the differences in SDM across age-groups than SA in this sample of adolescent football players. These findings suggest that the fluctuations in SDM could reflect the developmental challenges adolescents face, particularly during the adolescent growth spurt.^[19,20]^ This period is marked by rapid physical and neurological changes due to growth and maturation, rather than training-induced performance decrements.^[21]^ Such changes can temporarily disrupt basic motor skills and coordination, potentially impacting players’ self-perception and autonomy, a phenomenon known as the ‘adolescent awkwardness’ phase.^[19,20]^ Furthermore, the way peers, coaches, and support staff perceive and evaluate a player’s abilities can also influence SDM. The quality of relationships within the environment plays a significant role in shaping players’ perceived autonomy, underscoring the importance of fostering supportive environments that enhance positive self-perceptions and adaptive motivation during critical developmental stages. While our findings offer insight into psychological characteristics across age-groups, the poor inter-reliability of certain subscales may have introduced measurement noise, potentially influencing the observed patterns.

Although the study’s cross-sectional design limits causal conclusions, the observed differences across age-groups point towards the value of longitudinal research with appropriate measurement tools to track motivational changes over time. Regular assessments, perhaps biannually, could help monitor how motivation evolves, and guide tailored interventions to foster more autonomous motivation. By integrating CA, SA, and motivational orientation, practitioners can design development programs that foster sustained motivation and enhance overall well-being. Given the exploratory nature of this study, future research should aim to replicate these findings using instruments with higher reliability and cultural specificity that consider the athletes preferred language. Longitudinal tracking may also help improve measurement accuracy and reveal developmental patterns not captured in cross-sectional designs.

Coachability showed significant differences across age-groups, with younger players exhibiting lower values. These differences suggest that as players age, they become more receptive to instruction and constructive criticism, learning to distinguish between feedback and personal judgment. While both CA and SA had a moderate effect on coachability, neither was a dominant predictor when considered independently. This reinforces the idea that coachability is shaped by multiple factors beyond age, such as experience, training environments, and coaching approaches. As players progress, their ability to accept and apply feedback constructively becomes increasingly important, particularly in the tactical and strategic aspects of the game.^[3,11,18]^ Previous cross-sectional studies have suggested that individual differences in biological maturity may be associated with variations in players’ adaptive responses to coaching and developmental experiences.^[3,11]^ For example, a study of African junior football players found that late-maturing athletes scored higher than early-maturing players in coachability, as well as goal setting and mental preparation.^[3]^ Likewise, research on youth rugby players indicated that, over a two-year period, average-maturing players displayed improvements in coachability and general coping skills scores, while early-maturing players showed no change.^[11]^ While the rugby study had limitations, notably a small, selective sample size, it highlights how maturity may influence the development of coachability, although the relationship remains complex.^[11]^ Additionally, factors such as the quality of coach-athlete relationships, coaching styles, and the nature of feedback may contribute to the development of coachability and other psychological traits, emphasising the need for a broader exploration of these influences in future research.^[1,3,9]^

No significant linear relationships were observed between concentration and either CA or SA independently. However, when controlling for the other age measure, CA showed a weak positive association with concentration, while SA showed a weak negative association. This could suggest that as players progress in CA, their concentration may improve, whereas biologically advanced but younger players may struggle to maintain focus. These findings align with research indicating that late-maturing athletes tend to exhibit stronger concentration skills than early-maturing players, although the effect size was small.^[3]^ Concentration is a critical psychological skill that enables athletes to maintain focus under pressure and block distractions.^[3,11,18]^ These results highlight the importance of considering both CA and SA when assessing cognitive skills like concentration. They could suggest that late-maturing players may compensate for physical disadvantages by developing stronger focus, while early-maturing players may rely more on their physical advantages. ^[9,15]^ Further research is needed to explore how these factors interact to shape concentration, with implications for targeted training strategies.

Although few relationships were found between the psychological outcomes and age measures, our study provides valuable baseline data for research in this selective sample of football players from the Western Cape province. The lack of significant differences in some psychological traits may reflect the homogeneity of these academy players, where shared training environments and selection processes may contribute to similar psychological profiles. This suggests that within high-performance settings, key psychological skills may develop uniformly, regardless of age. Alternatively, it may indicate that meaningful differences in psychological characteristics only emerge at later developmental stages or in more diverse athlete populations. Progression to elite sport requires the development of psychological skills, which often evolve along individual pathways.^[1,5]^ However, significant gaps remain in understanding how these skills evolve from adolescence to adulthood and their role in long-term elite performance.^[2,5]^ Future studies should investigate whether these relationships stem from developmental factors or external influences, such as competition demands or challenges associated with physical maturation, to understand better how age and maturity impact concentration and coping skills.

The sample size, although appropriate for an exploratory study, was limited by the small number of participants per age-group, which restricted the likelihood of detecting significant differences between maturity status groups (i.e., early vs. late developers) or relative age-groups (e.g., birth quarter one vs. four). Although this is a minor consideration, it is worth noting that the study was not designed to identify such differences. Future studies with larger and more diverse samples are needed to conduct more robust statistical analyses and explore subgroup differences. Moreover, the psychological variables included in the study — such as those from the ACSI-28 — focused on specific skills tied to positive self-evaluations, which do not fully capture the participants’ broader psychological experiences. Future studies should utilise validated instruments that are suitable for use within the South African context. The low reliability observed in several subscales suggests potential inconsistencies in how participants responded to the items, limiting confidence in interpreting these findings. Consequently, any patterns of differences or associations involving these subscales must be considered exploratory and interpreted cautiously, as some findings may reflect measurement error or chance rather than true group differences or psychological characteristics. Similar concerns regarding inter-item reliability have been noted in studies of male African junior football players, which may affect the generalisability of the findings.^[3,4]^ To address these limitations, future research should validate these measures for cross-cultural and ecological relevance. Developing or adapting culturally appropriate tools with strong reliability and construct validity across developmental stages could contribute to more robust conclusions. A broader approach that considers factors such as training age, participation levels, and the coach-athlete relationship is also necessary. Incorporating diverse, reliable psychological assessment tools that reflect real-world experiences can enhance our understanding of how psychological skills develop and contribute to athletic performance.

## Conclusion

This study contributes to youth sport development literature by examining both CA and SA in relation to psychological characteristics among academy football players. Notably, the average SA in this sample was lower than the CA, with a significant trend towards later maturation overall. This contrasts with the typical selection bias towards early maturers generally seen in academy studies. The findings indicate that while certain motivational profiles and psychological skills demonstrate clear associations with CA or SA, other subscales lack significant relationships, highlighting the complex interplay between these variables. CA and SA are associated with variations in SDM, concentration, and coachability, with SDM showing a notable decline between chronological ages 15–17 before recovering in older players. Understanding both CA and SA can help practitioners identify fluctuations in self-determined motivation as players mature. Additionally, factors such as training age, participation involvement, and the coach-athlete relationship may significantly shape psychological characteristics.

These findings provide initial insights into how motivational and psychological characteristics vary across CA and SA groups. However, given the low reliability of several subscales, interpretations should be considered exploratory. Future research should utilise culturally validated, reliable instruments and longitudinal designs with larger, more diverse samples to better understand the evolving nature of psychological skills in youth athletes and their role in athletic development.

## Figures and Tables

**Table 1:** Internal consistency estimates (Cronbach’s alpha and Spearman-Brown coefficients) for various psychological subscales in South African adolescent football players (n=109)

Subscale	Cronbach’s Alpha (95% CI)	S-B Factor = 2	S-B Factor = 2.5	S-B Factor = 3	S-B Factor = 3.5	S-B Factor = 4

**SMS-II (18 items)**	.					
Amotivation	0.62 (0.47–0.72)*	0.77	0.81	0.84	0.86	0.88
External regulation	0.68 (0.52–0.78)	0.81	0.85	0.87	0.89	0.90
Introjected regulation	0.51 (0.32–0.65)*	0.68*	0.73	0.76	0.78	0.81
Identified regulation	0.58 (0.37–0.75)*	0.74	0.79	0.82	0.84	0.86
Integrated regulation	0.49 (0.28–0.64)*	0.65*	0.70	0.73	0.76	0.78
Intrinsic regulation	0.36 (0.12–0.53)*	0.53*	0.59*	0.63*	0.66*	0.69*

**ACSI-28 (28 items)**	.					
Coachability	0.56 (0.42–0.66)*	0.72	0.77	0.80	0.83	0.85
Concentration	0.23 (−0.04–0.44)*	0.38*	0.45*	0.50*	0.53*	0.56*
Confidence and achievement motivation	0.44 (0.21–0.59)*	0.61*	0.67*	0.71	0.74	0.76
Coping with adversity	0.35 (0.12–0.50)*	0.51*	0.57*	0.61*	0.65*	0.68*
Freedom from worry	0.62 (0.44–0.74)*	0.77	0.81	0.84	0.86	0.88
Goal setting and mental preparation	0.63 (0.50–0.73)*	0.77	0.81	0.84	0.86	0.88
Peaking under pressure	0.59 (0.39–0.72)*	0.74	0.79	0.82	0.84	0.86

S-B, Spearman-Brown;

* α < 0.70 (unacceptable);

Factor represents proportional increases in test length. Factor = 2, double test length; Factor 2.5, 2.5 times test length; Factor 3, 3 times test length; Factor 3.5, 3.5 times test length; Factor = 4, 4 times test length

**Table 2:** Summary statistics for body composition, age, and psychological characteristics in South African adolescent football players

Variable (n = 109)	Minimum	25th pct	Median	75th pct	Maximum	IQR	Mean±SD

Body mass (kg)	29.4	45.0	56.0	63.1	88.9	18.1	55.2±13.0
Height (cm)	135.1	155.6	166.4	172.2	189.5	16.6	163.8±12.1
BMI (kg/m^2^)	15.0	18.0	20.5	21.8	27.8	3.9	20.3±2.6
Chronological age (years)	9.9	12.7	15.0	16.7	18.9	4.0	14.9±2.4
Skeletal age (years)	9.3	12.7	14.7	16.0	18.2	3.2	14.3±2.2
SA-CA difference (years)	−3.0	−1.3	−0.5	0.3	1.9	1.7	−0.6±1.1

**SMS-II (n=109)**							
Amotivation	3.0	13.0	17.0	20.0	21.0	7.0	16.0±4.5
External regulation	3.0	12.0	16.0	19.0	21.0	7.0	15.1±4.8
Introjected regulation	3.0	8.0	10.0	13.0	18.0	5.0	10.2±4.0
Identified regulation	5.0	16.0	18.0	20.0	21.0	4.0	17.6±3.0
Integrated regulation	7.0	14.0	17.0	19.0	21.0	5.0	16.6±3.2
Intrinsic regulation	10.0	16.0	18.0	20.0	21.0	4.0	17.7±2.8
Relative autonomy index (RAI)	−39.0	−3.0	12.0	34.0	79.0	37.0	15.7±26.7

**ACSI-28 (n=107)**							
Coachability	3.0	8.0	10.0	11.0	12.0	3.0	9.3±2.4
Concentration	2.0	6.0	8.0	9.5	12.0	3.5	8.0±2.0
Confidence and achievement motivation	3.0	9.0	10.0	11.0	12.0	2.0	9.8±1.8
Coping with adversity	5.0	7.0	8.0	10.0	12.0	3.0	8.5±1.9
Freedom from worry	0.0	4.0	6.0	8.0	12.0	4.0	5.9±2.7
Goal setting and mental preparation	2.0	7.0	9.0	10.0	12.0	3.0	8.5±2.3
Peaking under pressure	0.0	6.0	8.0	9.0	12.0	3.0	7.7±2.4
ACSI-28 score	30.0	52.5	57.0	63.5	74.0	110	57.6±8.5

IQR, Interquartile range; pct, percentile; SD, Standard deviation

**Table 3:** Comparison of psychological characteristics across chronological age-groups in South African adolescent football players (n=109)

Variable	CA1	CA2	CA3	CA4	H Statistic	p-value	ɛ^2^

**SMS-II (n=109)**							
Amotivation	15.0±5.0	17.0±6.5	18.5±5.0	18.0±6.0	16.39	<0.001***	0.15
External regulation	14.0±6.0	16.0±8.0	18.0±4.0	15.0±9.0	11.82	0.001**	0.11
Introjected regulation	10.0±3.0	12.0±6.5	12.0±3.0	8.0±6.0	9.47	0.02*	0.09
Identified regulation	18.0±6.0	19.0±3.0	18.0±3.0	18.0±5.0	0.69	0.88	0.01
Integrated regulation	32.0±10.0	34.0±9.0	34.0±8.0	36.0±10.0	4.49	0.21	0.04
Intrinsic regulation	54.0±12.0	57.0±9.0	54.0±9.8	57.0±12.0	3.26	0.35	0.03
Relative autonomy index	29.0±40.0	7.0±42.5	0.0±18.3	18.0±26.0	10.92	0.01*	0.10

**ACSI-28 (n=107)**							
Coachability	8.0±2.0	11.0±3.5	10.5±4.0	11.0±3.5	8.12	0.04*	0.08
Concentration	8.0±3.0	9.0±2.5	8.0±3.3	8.0±3.0	6.10	0.11	0.06
Confidence and achievement motivation	10.0±3.0	10.0±2.0	10.0±3.3	10.0±2.0	0.89	0.83	0.01
Coping with adversity	8.0±2.0	9.0±2.0	8.0±2.0	9.0±2.5	4.61	0.20	0.04
Freedom from worry	6.0±4.0	6.0±3.0	6.0±3.5	6.0±2.5	0.91	0.82	0.01
Goal setting and mental preparation	8.0±4.0	9.0±2.5	9.0±3.0	10.0±2.0	3.18	0.36	0.03
Peaking under pressure	7.0±4.0	8.0±3.5	8.0±3.0	8.0±2.0	0.24	0.97	0.00
ACSI-28 score	53.0±10.0	59.0±12.5	58.5±10.5	59.0±12.0	6.49	0.09	0.06

* Indicates significance (p < 0.05),

** p < 0.01,

*** p < 0.001;

CA1–4, Chronological age-group 1–4; CA1, 9.9 to 12.7 years; CA2, 12.7 to 14.9 years; CA3, 14.9 to 16.7 years; CA4, 16.7 to 18.9 years; ɛ^2^, Epsilon squared effect size

**Table 4:** Comparison of psychological characteristics across skeletal age-groups in South African adolescent football players (n=109)

Variable	CA1	CA2	CA3	CA4	H Statistic	p-value	ɛ^2^

**SMS-II (n=109)**							
Amotivation	12.0±8.0	16.0±6.0	18.5±5.0	18.0±5.0	16.58	<0.001***	0.15
External regulation	14.0±7.0	18.0±7.0	19.0±4.8	15.0±6.5	13.10	<0.001***	0.12
Introjected regulation	10.0±4.0	11.0±5.0	11.0±5.3	10.0±6.5	1.16	0.76	0.01
Identified regulation	18.0±6.0	18.0±3.0	19.0±3.3	18.0±5.0	4.73	0.19	0.04
Integrated regulation	32.0±8.0	34.0±10.0	36.0±6.0	34.0±10.0	4.74	0.19	0.04
Intrinsic regulation	54.0±12.0	57.0±12.0	54.0±7.5	51.0±12.0	1.78	0.62	0.02
Relative autonomy index	25.0±44.0	7.0±41.0	7.0±23.0	14.0±34.5	4.48	0.21	0.04

**ACSI-28 (n=107)**							
Coachability	8.0±3.0	11.0±3.3	10.0±4.0	11.0±2.0	10.37	0.02*	0.10
Concentration	8.0±3.0	8.5±2.3	8.0±3.0	8.0±4.0	2.40	0.49	0.02
Confidence and achievement motivation	10.0±2.0	10.0±2.5	10.0±2.5	10.0±2.0	0.89	0.83	0.01
Coping with adversity	8.0±2.0	9.0±2.3	8.0±2.3	8.0±3.0	1.74	0.63	0.02
Freedom from worry	5.0±3.0	7.0±3.0	6.0±3.3	6.0±2.8	5.34	0.15	0.05
Goal setting and mental preparation	8.0±4.0	9.5±2.3	9.5±3.0	8.0±3.0	3.85	0.28	0.04
Peaking under pressure	8.0±4.0	6.5±3.0	8.0±3.3	8.0±1.8	3.23	0.36	0.03
ACSI-28 score	56.0±11.0	59.0±13.3	57.5±13.5	59.0±9.3	4.99	0.17	0.05

* Indicates significance (p < 0.05),

** p < 0.01,

*** p < 0.001;

CA1–4, Chronological age-group 1–4; CA1, 9.9 to 12.7 years; CA2, 12.7 to 14.9 years; CA3, 14.9 to 16.7 years; CA4, 16.7 to 18.9 years; ɛ^2^, Epsilon squared effect size

**Table 5:** Pairwise comparisons of SMS-II subscales across CA and SA groups in South African adolescent football players (n=109)

Chronological age (CA)					Skeletal age (SA)			

Variables	Comparison	Statistic	p-value	r_rb_	Comparison	Statistic	p-value	r_rb_

Amotivation	CA1 vs CA2	237.0	0.08	−0.29	SA1 vs SA2	283.0	0.03*	−0.33
	CA1 vs CA3	196.0	<0.001***	−0.52	SA1 vs SA3	188.5	<0.001***	−0.54
	CA1 vs CA4	203.0	<0.001***	−0.52	SA1 vs SA4	172.0	<0.01**	−0.48
	CA2 vs CA3	234.0	0.10	−0.27	SA2 vs SA3	275.5	0.04*	−0.32
	CA2 vs CA4	245.0	0.10	−0.27	SA2 vs SA4	265.0	0.21	−0.21
	CA3 vs CA4	418.5	0.84	0.03	SA3 vs SA4	368.0	0.38	0.14

External	CA1 vs CA2	236.0	0.07	−0.29	SA1 vs SA2	276.0	0.03*	−0.34
	CA1 vs CA3	177.0	<0.001***	−0.56	SA1 vs SA3	224.0	<0.01**	−0.45
	CA1 vs CA4	344.0	0.23	−0.18	SA1 vs SA4	301.0	0.55	−0.10
	CA2 vs CA3	255.0	0.20	−0.21	SA2 vs SA3	343.5	0.32	−0.15
	CA2 vs CA4	353.5	0.72	0.06	SA2 vs SA4	435.5	0.06	0.31
	CA3 vs CA4	516.5	0.08	0.27	SA3 vs SA4	466.0	<0.01**	0.45

Introjected	CA1 vs CA2	275.5	0.29	−0.17	SA1 vs SA2	416.5	0.96	−0.01
	CA1 vs CA3	246.5	0.01*	−0.39	SA1 vs SA3	356.0	0.43	−0.12
	CA1 vs CA4	474.0	0.41	0.13	SA1 vs SA4	359.5	0.64	0.08
	CA2 vs CA3	286.5	0.51	−0.11	SA2 vs SA3	366.0	0.53	−0.10
	CA2 vs CA4	414.5	0.14	0.24	SA2 vs SA4	355.5	0.69	0.07
	CA3 vs CA4	577.0	<0.01**	0.42	SA3 vs SA4	372.0	0.35	0.16

Identified	CA1 vs CA2	325.5	0.89	−0.02	SA1 vs SA2	434.5	0.83	0.03
	CA1 vs CA3	394.5	0.86	−0.03	SA1 vs SA3	321.0	0.17	−0.21
	CA1 vs CA4	376.5	0.49	−0.10	SA1 vs SA4	353.5	0.72	0.06
	CA2 vs CA3	324.0	0.98	0.01	SA2 vs SA3	284.5	0.05	−0.30
	CA2 vs CA4	299.5	0.53	−0.10	SA2 vs SA4	346.0	0.82	0.04
	CA3 vs CA4	366.0	0.52	−0.10	SA3 vs SA4	419.0	0.06	0.30

Integrated	CA1 vs CA2	287.5	0.40	−0.14	SA1 vs SA2	369.0	0.42	−0.12
	CA1 vs CA3	346.0	0.34	−0.15	SA1 vs SA3	274.0	0.04*	−0.33
	CA1 vs CA4	290.0	0.04*	−0.31	SA1 vs SA4	264.0	0.2	−0.21
	CA2 vs CA3	328.0	0.92	0.02	SA2 vs SA3	325.5	0.2	−0.20
	CA2 vs CA4	272.5	0.26	−0.18	SA2 vs SA4	302.0	0.57	−0.09
	CA3 vs CA4	329.0	0.22	−0.19	SA3 vs SA4	355.0	0.54	0.10

Intrinsic	CA1 vs CA2	322.5	0.84	−0.03	SA1 vs SA2	399.5	0.75	−0.05
	CA1 vs CA3	459.5	0.39	0.13	SA1 vs SA3	346.5	0.34	−0.15
	CA1 vs CA4	365.0	0.39	−0.13	SA1 vs SA4	349.0	0.78	0.05
	CA2 vs CA3	388.5	0.21	0.21	SA2 vs SA3	373.5	0.60	−0.08
	CA2 vs CA4	302.5	0.57	−0.09	SA2 vs SA4	369.0	0.51	0.11
	CA3 vs CA4	300.0	0.09	−0.26	SA3 vs SA4	392.5	0.18	0.22

Relative autonomy index	CA1 vs CA2	396.0	0.25	0.19	SA1 vs SA2	506.5	0.18	0.20
	CA1 vs CA3	608.5	<0.01**	0.50	SA1 vs SA3	529.5	0.05	0.30
	CA1 vs CA4	512.0	0.16	0.22	SA1 vs SA4	414.5	0.14	0.24
	CA2 vs CA3	388.5	0.21	0.21	SA2 vs SA3	438.5	0.61	0.08
	CA2 vs CA4	320.5	0.82	−0.04	SA2 vs SA4	332.0	0.98	0.00
	CA3 vs CA4	254.0	0.02*	−0.37	SA3 vs SA4	280.5	0.44	−0.13

* Indicates significance (p < 0.05),

** p < 0.01,

*** p < 0.001;

statistic, Wilcoxon rank-sum test; r_rb,_ rank biserial correlation

**Table 6:** Pairwise comparisons of ACSI-28 subscales across CA and SA groups in South African adolescent football players (n=107)

	Chronological age (CA)				Skeletal age (SA)			

Variables	Comparison	Statistic	p-value	r_rb_	Comparison	Statistic	p-value	r_rb_

Coachability	CA1 vs CA2	234.0	0.07	−0.30	SA1 vs SA2	233.5	<0.01**	−0.42
	CA1 vs CA3	258.5	0.02*	−0.36	SA1 vs SA3	266.5	0.03*	−0.34
	CA1 vs CA4	242.0	0.01*	−0.38	SA1 vs SA4	187.0	0.01*	−0.41
	CA2 vs CA3	315.0	0.90	−0.02	SA2 vs SA3	424.5	0.59	0.08
	CA2 vs CA4	288.5	0.67	−0.07	SA2 vs SA4	344.0	0.48	0.12
	CA3 vs CA4	371.0	0.91	−0.02	SA3 vs SA4	310.0	0.98	0.01

Concentration	CA1 vs CA2	213.5	0.03*	−0.36	SA1 vs SA2	311.0	0.13	−0.23
	CA1 vs CA3	388.0	0.78	−0.04	SA1 vs SA3	368.0	0.54	−0.09
	CA1 vs CA4	308.5	0.17	−0.21	SA1 vs SA4	307.5	0.83	−0.04
	CA2 vs CA3	420.5	0.06	0.31	SA2 vs SA3	444.5	0.39	0.13
	CA2 vs CA4	355.0	0.39	0.14	SA2 vs SA4	358.0	0.33	0.16
	CA3 vs CA4	318.5	0.32	−0.16	SA3 vs SA4	326.0.	0.73	0.06

Confidence and achievement motivation	CA1 vs CA2	348.5	0.78	0.04	SA1 vs SA2	377.5	0.65	−0.07
	CA1 vs CA3	374.5	0.61	−0.08	SA1 vs SA3	344.0	0.31	−0.15
	CA1 vs CA4	369.5	0.72	−0.06	SA1 vs SA4	301.5	0.74	−0.05
	CA2 vs CA3	283.5	0.46	−0.12	SA2 vs SA3	370.5	0.73	−0.05
	CA2 vs CA4	265.0	0.37	−0.15	SA2 vs SA4	305.0	0.96	−0.01
	CA3 vs CA4	382.5	0.94	0.01	SA3 vs SA4	332.0	0.64	0.08

Coping with adversity	CA1 vs CA2	276.0	0.29	−0.17	SA1 vs SA2	332.5	0.24	−0.18
	CA1 vs CA3	460.0	0.39	0.13	SA1 vs SA3	415.0	0.89	0.02
	CA1 vs CA4	328.0	0.30	−0.16	SA1 vs SA4	300.5	0.73	−0.06
	CA2 vs CA3	416.0	0.07	0.29	SA2 vs SA3	460.0	0.26	0.17
	CA2 vs CA4	301.0	0.86	−0.03	SA2 vs SA4	338.5	0.55	0.10
	CA3 vs CA4	278.0	0.09	−0.26	SA3 vs SA4	287.5	0.69	−0.07

Freedom from worry	CA1 vs CA2	286.5	0.39	−0.14	SA1 vs SA2	310.5	0.13	−0.24
	CA1 vs CA3	367.0	0.54	−0.10	SA1 vs SA3	412.0	0.93	0.01
	CA1 vs CA4	359.0	0.60	−0.08	SA1 vs SA4	229.0	0.09	−0.28
	CA2 vs CA3	328.0	0.92	0.02	SA2 vs SA3	487.0	0.12	0.24
	CA2 vs CA4	341.0	0.55	0.10	SA2 vs SA4	302.5	0.92	−0.02
	CA3 vs CA4	390.0	0.84	0.03	SA3 vs SA4	223.0	0.10	−0.28

Goal setting and mental preparation	CA1 vs CA2	271.5	0.25	−0.19	SA1 vs SA2	307.5	0.11	−0.24
	CA1 vs CA3	340.0	0.29	−0.16	SA1 vs SA3	304.0	0.10	−0.25
	CA1 vs CA4	293.0	0.10	−0.25	SA1 vs SA4	283.5	0.50	−0.11
	CA2 vs CA3	329.0	0.90	0.02	SA2 vs SA3	386.0	0.93	−0.02
	CA2 vs CA4	275.5	0.50	−0.11	SA2 vs SA4	353.0	0.38	0.15
	CA3 vs CA4	334.0	0.46	−0.12	SA3 vs SA4	356.0	0.35	0.16

Peaking under pressure	CA1 vs CA2	331.0	0.97	−0.01	SA1 vs SA2	487.0	0.20	0.20
	CA1 vs CA3	397.0	0.89	−0.02	SA1 vs SA3	385.0	0.74	−0.05
	CA1 vs CA4	363.5	0.65	−0.07	SA1 vs SA4	333.5	0.79	0.05
	CA2 vs CA3	324.5	0.97	0.01	SA2 vs SA3	295.5	0.11	−0.25
	CA2 vs CA4	299.0	0.83	−0.04	SA2 vs SA4	260.5	0.35	−0.15
	CA3 vs CA4	353.0	0.68	−0.07	SA3 vs SA4	360.5	0.30	0.17

ACSI-28 score	CA1 vs CA2	226.0	0.05	−0.32	SA1 vs SA2	275.5	0.04*	−0.32
	CA1 vs CA3	310.5	0.13	−0.24	SA1 vs SA3	311.0	0.13	−0.23
	CA1 vs CA4	249.5	0.02*	−0.36	SA1 vs SA4	235.5	0.11	−0.26
	CA2 vs CA3	354.5	0.54	0.10	SA2 vs SA3	413.0	0.74	0.05
	CA2 vs CA4	309.5	0.99	0.00	SA2 vs SA4	338.0	0.56	0.10
	CA3 vs CA4	335.5	0.48	−0.11	SA3 vs SA4	304.0	0.94	−0.01

* Indicates significance (p < 0.05),

** p < 0.01,

*** p < 0.001;

statistic, Wilcoxon rank-sum test; r_rb,_ rank biserial correlation

**Table 7:** Spearman’s correlation analysis of CA and SA with psychological characteristics in South African adolescent football players (n=109)

Chronological age (CA)					Skeletal age (SA)			

Outcome variables	Rho (95% CI)	p-value	Rho partial	p-value partial	Rho (95% CI)	p-value	Rho partial	p-value partial

**SMS-II (n=109)**								
Amotivation	0.38 (0.20; 0.55)	<0.001***	0.05	0.61	0.41 (0.24; 0.56)	<0.001***	0.18	0.06
External regulation	0.20 (0.01; 0.39)	0.04*	0.14	0.16	0.15 (−0.04; 0.32)	0.12	0.05	0.64
Introjected regulation	0.01 (−0.18; 0.21)	0.92	−0.07	0.47	0.05 (−0.13; 0.25)	0.60	0.09	0.37
Identified regulation	0.06 (−0.14; 0.24)	0.57	0.09	0.37	0.01 (−0.18; 0.21)	0.88	−0.07	0.48
Integrated regulation	0.19 (−0.01; 0.36)	0.05*	0.08	0.43	0.18 (−0.02; 0.36)	0.07	0.02	0.83
Intrinsic regulation	0.06 (−0.14; 0.26)	0.52	0.09	0.38	0.02 (−0.15; 0.22)	0.82	−0.06	0.51
Relative autonomy index	−0.18 (−0.36; 0.01)	0.07	0.01	0.88	−0.21 (−0.39; −0.01)	0.03*	−0.12	0.23

**ACSI-28 (n=107)**								
Coachability	0.24 (0.06; 0.4)	0.01**	0.07	0.46	0.24 (0.07; 0.41)	0.01**	0.06	0.56
Concentration	0.09 (−0.09; 0.29)	0.35	0.24	0.01**	−0.03 (−0.20; 0.17)	0.77	−0.22	0.02*
Confidence and achievement motivation	0.02 (−0.18; 0.21)	0.87	−0.01	0.93	0.02 (−0.16; 0.19)	0.81	0.02	0.84
Coping with adversity	0.05 (−0.15; 0.24)	0.64	0.11	0.24	−0.01 (−0.18; 0.16)	0.91	−0.11	0.28
Freedom from worry	0.06 (−0.14; 0.26)	0.52	−0.02	0.82	0.08 (−0.10; 0.27)	0.39	0.06	0.54
Goal setting and mental preparation	0.12 (−0.07; 0.30)	0.22	0.12	0.23	0.07 (−0.13; 0.26)	0.46	−0.07	0.49
Peaking under pressure	0.04 (−0.17; 0.25)	0.66	0.12	0.27	−0.02 (−0.20; 0.17)	0.84	−0.12	0.24
ACSI-28 score	0.18 (0.00; 0.36)	0.06	0.17	0.09	0.11 (−0.06; 0.27)	0.25	−0.09	0.36

* Indicates significance (p < 0.05),

** p < 0.01,

*** p < 0.001;

Rho, Spearman correlation

## References

[1] Musculus L Lobinger BH Psychological characteristics in talented soccer players – recommendations on how to improve coaches’ assessment Front Psychol 2018 9 1 6 10.3389/fpsyg.2018.00041 29459839 PMC5807374

[2] Toering T Elferink-Gemser M Jordet G Pepping G Visscher C Self-regulation of learning and performance level of elite youth soccer players Int J Sport Psychol 2012 43 4 1 14 10.1080/02640410903369919 19967593

[3] Van Den Berg L Grobbelaar HW Jooste J Jacobs S Psychological factors may counterbalance physical disadvantage of late maturation among African junior soccer players S Afr J Res Sport, Physic Edu and Recreat 2019 41 3 117 127

[4] Jooste J Steyn BJM Van Den Berg L Psychological skills, playing positions and performance of African youth soccer teams S Afr J Res Sport, Physic Edu and Recreat 2014 36 1 85 100

[5] Van Yperen NW Why some make it and others do not: Identifying psychological factors that predict career success in professional adult soccer Sport Psychol 2009 23 3 317 329 [10.1123/tsp.23.3.317]

[6] Salmon J Hall C Haslam I The use of imagery by soccer players J Appl Sport Psychol 1992 6 1 116 133 [10.1080/10413209408406469]

[7] Lloyd RS Oliver JL Faigenbaum AD Myer GD De Ste Croix MBA Chronological age vs. biological maturation: implications for exercise programming in youth J Strength Cond Res 2014 28 5 1454 1464 10.1519/JSC.0000000000000391 24476778

[8] Cumming SP Searle C Hemsley JK Biological maturation, relative age and self-regulation in male professional academy soccer players: A test of the underdog hypothesis Psychol Sport Exerc 2018 39 147 153 [10.1016/j.psychsport.2018.08.007]

[9] Hancock DJ Adler AL Côté J A proposed theoretical model to explain relative age effects in sport Eur J Sport Sci 2013 13 6 630 637 10.1080/17461391.2013.775352 24251740

[10] Ruf L Cumming S Härtel S Hecksteden A Drust B Meyer T Construct validity of percentage of predicted adult height and BAUS skeletal age to assess biological maturity in academy soccer Ann Hum Biol 2021 48 2 101 109 10.1080/03014460.2021.1913224 34097548

[11] Van den Berg L Pienaar AE Grobbelaar HW The role of biological maturity in sport psychological skills of young rugby players: an explorative investigation Afr J Phys Health Educ Recreat Dance 2012 18 332 343

[12] Hendry DT Crocker PRE Williams AM Hodges NJ Tracking and comparing self-determined motivation in elite youth soccer: influence of developmental activities, age, and skill Front Psychol 2019 10 304 10.3389/fpsyg.2019.00304 30890977 PMC6411691

[13] Self-Determination Theory: basic psychological needs in motivation, development, and wellness New York Guilford Publications 2017 10.1521/978.14625/28806]

[14] Pelletier LG Rocchi MA Vallerand RJ Deci EL Ryan RM Validation of the revised sport motivation scale (SMS-II) Psychol Sport Exerc 2013 14 3 329 341 [10.1016/j.psychsport.2012.12.002]

[15] Hill M Spencer A McGee D Scott S Frame M Cumming SP The psychology of bio-banding: A Vygotskian perspective Ann Hum Biol 2020 47 4 328 335 10.1080/03014460.2020.1797163 32674664

[16] Calvo TG Cervelló E Jiménez R Iglesias D Murcia JAM Using self-determination theory to explain sport persistence and dropout in adolescent athletes Span J Psychol 2010 13 2 677 684 10.1017/s1138741600002341 20977017

[17] Rachmiel M Naugolni L Mazor-Aronovitch K Koren-Morag N Bistritzer T Bone age assessments by quantitative ultrasound (SonicBone) and hand X-ray based methods are comparable Isr Med Assoc J 2017 19 9 533 537 28971634

[18] Smith RE Schutz RW Smoll FL Ptacek JT Development and validation of a multidimensional measure of sport-specific psychological skills: The Athletic Coping Skills Inventory-28 J Sport Exerc Psychol 1995 17 4 379 398 [10.1123/jsep.17.4.379]

[19] Quatman-Yates CC Quatman CE Meszaros AJ Paterno MV Hewett TE A systematic review of sensorimotor function during adolescence: a developmental stage of increased motor awkwardness? Br J Sports Med 2012 46 9 649 655 10.1136/bjsm.2010.079616 21459874 PMC4157222

[20] Pichardo AW Oliver JL Harrison CB Maulder PS Lloyd RS Kandoi R The Influence of maturity offset, strength, and movement competency on motor skill performance in adolescent males Sports (Basel) 2019 7 7 168 [10.3390/sports7070168 31323944 PMC6680597

